# A Cancer Specific Cell-Penetrating Peptide, BR2, for the Efficient Delivery of an scFv into Cancer Cells

**DOI:** 10.1371/journal.pone.0066084

**Published:** 2013-06-11

**Authors:** Ki Jung Lim, Bong Hyun Sung, Ju Ri Shin, Young Woong Lee, Da Jung Kim, Kyung Seok Yang, Sun Chang Kim

**Affiliations:** 1 Department of Biological Sciences, Korea Advanced Institute of Science and Technology, Daejeon, Korea; 2 Biochemicals and Synthetic Biology Research Center, Korea Research Institute of Bioscience and Biotechnology, Daejeon, Korea; University of Pittsburgh, United States of America

## Abstract

Cell-penetrating peptides (CPPs) have proven very effective as intracellular delivery vehicles for various therapeutics. However, there are some concerns about non-specific penetration and cytotoxicity of CPPs for effective cancer treatments. Herein, based on the cell-penetrating motif of an anticancer peptide, buforin IIb, we designed several CPP derivatives with cancer cell specificity. Among the derivatives, a 17-amino acid peptide (BR2) was found to have cancer-specificity without toxicity to normal cells. After specifically targeting cancer cells through interaction with gangliosides, BR2 entered cells via lipid-mediated macropinocytosis. Moreover, BR2 showed higher membrane translocation efficiency than the well-known CPP Tat (49–57). The capability of BR2 as a cancer-specific drug carrier was demonstrated by fusion of BR2 to a single-chain variable fragment (scFv) directed toward a mutated K-ras (G12V). BR2-fused scFv induced a higher degree of apoptosis than Tat-fused scFv in K-ras mutated HCT116 cells. These results suggest that the novel cell-penetrating peptide BR2 has great potential as a useful drug delivery carrier with cancer cell specificity.

## Introduction

The beneficial effects of many newly-discovered potential therapeutic agents, such as proteins, nucleic acids, and hydrophilic drugs, are limited because of their inability to reach the appropriate intracellular targets [1], [2]. Thus, numerous approaches such as microinjection, eletroporation, liposomal formulation and the use of viral vectors have been explored to promote efficient drug delivery [3], [4]. One major concern about these techniques is their poor cell specificity [4], [5]. Therefore, the development of a target-specific drug delivery system is a primary concern for improving the therapeutic efficacy of drugs while reducing their effective doses and side effects [6], [7].

Cell-penetrating peptides (CPPs), also referred to as protein transduction domains, have drawn special attention as an alternative intracellular drug delivery vehicle since the discovery of the first CPP, Tat, in 1988 [8]. CPPs are short peptides consisting of fewer than 30 amino acids and composed mostly of basic, positively charged amino acids (e.g. Arg, Lys and His) that have the capacity to translocate through the cell membrane and to deliver a variety of cell-impermeable cargoes across the cellular membrane [9], including proteins [10], nucleic acids [11], siRNA [12], peptide nucleic acids (PNAs) [13], small molecule therapeutics [14], quantum dots [15], and MRI contrast agents [16]. Although the exact mechanism of CPPs is unknown, recent mechanistic studies imply that their cellular uptake results from an initial rapid electrostatic interaction with the plasma membrane followed by endosomal uptake [17], [18].

Using CPPs for the intracellular delivery of a wide range of macromolecules is a powerful approach because of their versatility paired with easy functionalization of linked cargoes and the high delivery efficiency into various cell lines, overcoming challenges often faced with other delivery methods [19], [20]. Therefore, many studies have focused on the development of novel CPPs; the number of available CPPs with different characteristics, such as increased stability and efficient cargo delivery, continues to increase [17].

Although the potential of CPPs as delivery agents is large, their lack of cell specificity, cytotoxic effects and unexpected side effects are major concerns for their development as drug delivery vehicles [21]. For cancer therapy, CPP cell specificity is especially important so that side effects on normal cells are minimized [22], [23]. Therefore, there is a strong need for the development of cancer-specific and non-toxic CPPs for effective cancer treatments.

We have previously reported that a potent antimicrobial peptide, buforin IIb (RAGLQFPVG[RLLR]_3_), has strong cell-penetrating ability and anticancer activity against various cancer cell lines [24], [25]. Even though buforin IIb showed selective cytotoxicity against cancer cells, it also affected the viability of normal cells at high concentrations. To develop buforin IIb as an efficient drug delivery vehicle, its cytotoxicity against normal cells should be minimized while maintaining its cancer cell specificity.

In this study, we designed a novel cancer-specific and non-toxic cell-penetrating peptide, BR2, based on the cell-penetrating motif of buforin IIb and studied the potential as an efficient drug delivery vehicle into cancer cells by fusing BR2 to a single-chain variable fragment (scFv) antibody against mutated K-ras.

## Materials and Methods

### Cell Culture

Human cervical cancer cell line HeLa, human colon cancer cell line HCT116, mouse melanoma cell line B16/F10, mouse fibroblast cell line NIH 3T3, human keratinocyte cell line HaCat and human fibroblast cell line BJ were all obtained from American Type Culture Collection (ATCC; Manassas, VA) and cultured in a complete medium [Dulbecco’s modified eagle medium] (DMEM) supplemented with 10% fetal bovine serum (FBS), 100 units/ml penicillin, 100 µg/ml streptomycin. Cells were grown in humidified conditions at 37°C with 5% CO_2_.

### Peptide Design and Synthesis

We designed several derivatives of buforin IIb (BR3) by stepwise elimination of the C-terminal regular α-helical motif RLLR repeats of buforin IIb to create a cancer cell specific and non-toxic CPP. The designed peptides consisted of different numbers of the C-terminal regular α-helical motif RLLR and named BR1 and BR2 (Table 1).

**Table 1:** Amino acid sequences of peptides used in this study.

*Peptides*	*Amino acid sequence*	*Charge*	*Ref.*
Tat (49–57)	RKKRRQRRR (9 aa)	+8	[46]
Buf IIb [BR3]	RAGLQFPVGRLLRRLLRRLLR (21 aa)	+7	[47]
BR2	RAGLQFPVGRLLRRLLR (17 aa)	+5	This study
BR1	RAGLQFPVGRLLR (13 aa)	+3	This study

Underline indicates the model α-helical sequence.

CPPs Tat, BR1, BR2, and BR3 were chemically synthesized (Anygen, Kwangju, Korea) on a MilliGen 9050 peptide synthesizer. The fluorescein moiety (FITC) was attached to the N-terminus via an aminohexanoic acid spacer by treating a resin-bound peptide (0.1 mM) with FITC (0.1 mM) and diisopropyl ethyl amine (0.5 mM) in N, N-Dimethylformamide (DMF) for 12 h. All crude peptides were purified and analyzed by reversed-phase high performance liquid chromatography (RP-HPLC) on a C18 column, and the purified peptides were characterized by electrospray ionization mass spectrometry (ESI-MS).

### Confocal Laser Scanning Microscopy

To investigate the cell-penetrating ability and the intracellular distribution of the internalized peptides, live confocal microscopy was performed on three cancer lines (HeLa, HCT116 and B16/F10) and three normal cell lines (HaCat, BJ and NIH 3T3). Briefly, cells (2×10^5^) were plated on a glass coverslip placed in a 6-well plate, grown overnight, and then incubated with FITC-labeled peptides (5 µM for each cell line) for 30 min. The cells were then rinsed three times with phosphate buffered saline (PBS, pH 7.4), and mounted on microscope slides with fluorescence mounting solution (Dako Corp, Carpinteria, CA). Colocalization of BR2 with lysosomes was observed by using LysoTracker Red DND-99 (Molecular Probe, Eugene, OR). To avoid the effects of fixation artifacts, involving both methanol and paraformaldehyde, cells were not fixed [26], [27]. The distribution of FITC-labeled peptides was analyzed using a confocal scanning laser Zeiss LSM 510 microscope (Jena, Germany) equipped with a 40× and 20× objective. Fluorophores were excited with an argon laser (488 nm) for FITC and a HeNe laser (543 nm) for LysoTracker Red.

### *In vitro* Cytotoxicity Assay

The cytotoxicity of peptides to mammalian cells was investigated by assessing the release of lactate dehydrogenase (LDH) from cancer and normal cells. The amount of LDH released from damaged cells into the supernatant was measured using the Cytotoxicity Detection Kit (Roche Applied Science, Germany) according to the manufacturer’s instructions. In brief, cells were plated onto 96-well microplates (1×10^4^ cells per well) in complete DMEM supplemented with 10% FBS and incubated overnight at 37°C to allow for attachment and spreading of cells. After 24 h of incubation, cells were treated with various concentrations of peptides (0–100 µM) and incubated for another 24 h at 37°C. The extracellular medium from each well was transferred to a new microplate and incubated for 10 min with 100 µl/well reaction mixture, followed by a stop solution. Absorbance was measured at 490 nm by using an ELISA plate reader. LDH release from cells lysed with 0.2% Triton X-100 in PBS was defined as 100% leakage and LDH release from untreated cells as 0% leakage.

### Hemolysis Assay

Hemolytic activity was assayed as described by Aboudy *et al.* with slight modifications [28]; 3 ml of freshly prepared human erythrocytes was washed with isotonic PBS, pH 7.4, until the color of the supernatant turned clear. The washed erythrocytes were then diluted to a final volume of 20 ml with the same buffer. Peptide samples (10 µl), serially diluted in PBS, were added to 190 µl of the cell suspension in microcentrifuge tubes. Following gentle mixing, the tubes were incubated at 37°C for 30 min and then centrifuged at 4,000×g for 5 min. The supernatant (100 µl) was removed to a new tube and the absorbance at 567 nm was determined. The relative optical density, as compared with that of the cell suspension treated with 0.2% Triton X-100, was defined as percentage of hemolysis. The hemolysis percentage was calculated using the following equation: percentage hemolysis = [(Abs _567 nm_ in the peptide solution – Abs _567 nm_ in PBS)/(Abs _567 nm_ in 0.2% Triton X-100 – Abs _567 nm_ in PBS)]×100.

### Characterization of Peptide Uptake

To evaluate the internalization of FITC-labeled peptides, HeLa cells were seeded onto 12-well plates at a density of 2×10^5^ cells per well and incubated for 24 h. FITC-labeled peptides, at various concentrations ranging from 2 to 10 µM, were then incubated with the cells for 30 min at 37°C. To compare the cellular uptake of peptides, cancer and normal cells were treated with FITC-labeled peptides (each, 10 µM) and incubated for 30 min at 37°C. Following the incubation, cells were washed three times with ice-cold PBS to remove excess extracellular complexes. Next, the cells were treated with trypsin (1 mg/ml) for 10 min to remove any remaining peptides bound to the cell surface. After trypsinization, the cells were collected by centrifugation (1,000×g for 5 min), resuspended with 500 µl ice-cold 2% FBS/PBS containing propidium iodide (PI), and then immediately analyzed (10,000 events/sample) by fluorescence activated cell sorting (FACS).

To understand further the cell-penetrating mechanism of peptides, the effects of temperature and metabolic inhibitors were examined. To elucidate the temperature dependency, HeLa cells were incubated at 4°C for 30 min prior to the addition of the peptides. Next, cells were treated with FITC-labeled peptides (each, 5 µM) at 4°C for 30 min. For the energy-depletion study, HeLa cells were preincubated with sodium azide (NaN_3,_ 10 mM) at 37°C for 1 h and then incubated with FITC-labeled peptides (each, 5 µM) at 37°C for 30 min.

To study the role of endocytosis in peptide uptake, cells were pretreated with several endocytosis inhibitors at 37°C for 1 h: (i) amiloride (5 mM), which is known to block macropinocytosis by inhibiting a sodium channel; (ii) nocodazole (100 ng/ml), which inhibits the clathrin-mediated pathway; and (iii) methyl-ß-cyclodextrin (MßCD, 5 mM), which inhibits lipid raft-mediated processes by depleting cholesterol from the plasma membrane [29]. All inhibitors were purchased from Sigma (St. Louis, MO).

To examine the effects of negatively charged components on the cell surface for peptide internalization, HeLa cells were pretreated with gangliosides (monosialoganglioside GM3 from canine blood), heparin sulfate, or sialic acid (Neu5Ac, all from Sigma) (20 µg/ml) for 30 min. For the inhibition of ganglioside biosynthesis, cells were pretreated with D-*threo*-1-phenyl-2-hexadecanoyl amino-3-morpholino-1-propanol (PPMP, 5 µM) for 48 h.

For all of these experimental conditions, flow cytometry analyses were performed with live cells using a Becton Dickinson FACSCalibur flow cytometer (BD Biosciences, San Diego, CA). In each case, the fluorescence of 10,000 viable cells was acquired. Viable cells were gated based on a sideward and forward scatter. For data analysis, WinMDI software (Joe Trotter, Scripps Research Institute, La Jolla, CA) was used. The statistical significance was evaluated by Student’s t-test at a 95% confidence interval.

### Cloning and Expression of Peptide-fusion Proteins

To employ BR2 as a vehicle for the delivery of therapeutic proteins, a cDNA of the Y13–259 single-chain variable fragment (scFv) gene was synthesized at Bioneer (Daejeon, Korea). The Tat- or BR2-fused Y13–259 scFv genes were obtained by recombinant PCR. The recombinant cDNAs encoding the anti-Ras scFv, Tat-scFv and BR2-scFv fusions were digested with *Nco*I and *EcoR*I (both from New England Biolabs, Beverly, MA), and cloned into the *Nco*I and *EcoR*I sites of pET21c, producing pscFv, pTat-scFv and pBR2-scFv, respectively. Anti-Ras scFv, Tat-scFv and BR2-scFv fusion proteins were expressed in *E. coli* Origami (DE3) after induction with 0.1 mM IPTG for 4 h at 37°C. Cells were harvested by centrifugation at 3,000×g for 15 min at 4°C. The cell pellet was resuspended in phosphate buffer (10 mM sodium phosphate, 150 mM NaCl, pH 7.4); cells were disrupted by sonication at 4°C (B. Braun instruments, Allentown, PA). The protease inhibitor phenylmethylsulfonyl fluoride (PMSF, 1 mM) was added prior to sonication. The soluble and insoluble fractions were separated by centrifugation at 14,000×g for 15 min at 4°C. The pellet containing the inclusion bodies was resuspended in wash buffer (20 mM Tris–HCl, 5 mM EDTA and 1% Triton X-100, pH 8.0) and centrifuged at 8,000×g for 10 min at 4°C.

The washed inclusion bodies were denatured and solubilized in lysis buffer (0.3% N-lauroyl sarcosine, 50 mM CAPS buffer, and 0.3 M NaCl, pH 11.0) for 3 h and centrifuged at 14,000×g for 15 min at 4°C. All proteins were affinity-purified by using the Ni–IDA agarose resin (ELPIS biotech, Daejeon, Korea). In brief, the Ni-IDA His-Bind Resin was packed into a column equilibrated with binding buffer (identical to lysis buffer). The supernatant was slowly applied to the column, after which wash buffer (0.3% N-lauroyl sarcosine, 50 mM CAPS buffer, 150 mM NaCl, and 30 mM imidazole, pH 11.0) was applied. The proteins were eluted with elution buffer (0.3% N-lauroyl sarcosine, 50 mM CAPS buffer, 150 mM NaCl, and 250 mM imidazole, pH 11.0) and analyzed by 10% SDS–PAGE. The eluted proteins were refolded by dialysis in PBS containing 200 mM NaCl, 10% glycerol, 1 mM GSH, 0.2 mM GSSG and 0.4 M arginine with gradual pH reduction (pH 10, pH 9, pH 8 and pH 7.4). Each dialysis step was performed at 4°C for 12 h against 20× sample volume to remove the detergent completely.

### Western Blotting

To monitor the peptide-mediated intracellular uptake of scFv proteins, the internalized fusion proteins were examined by Western blotting. HCT116 cells (1×10^6^) were treated with PBS, purified scFv, Tat-scFv, or BR2-scFv fusion proteins (each, 2 µM) for 2 h at 37°C. The cells were then washed twice with PBS (4°C), scraped into 0.5 ml of PBS and centrifuged at 1,000×g at 4°C for 5 min. The cell pellets were resuspended in 100 µl of lysis buffer (20 mM Tris-HCl (pH 7.5), 150 mM NaCl, 1 mM Na_2_EDTA, 1 mM EGTA, 1% Triton, 2.5 mM sodium pyrophosphate, 1 mM beta-glycerophosphate, 1 mM Na_3_VO_4_, 1 µg/ml leupeptin and 1 mM PMSF (Cell Signaling Tech., Danvers, MA) and kept on ice for 30 min. The cell lysates were then centrifuged at 12,000×g at 4°C for 15 min and the supernatants were collected. Protein concentrations in the cell extracts were determined by the Bradford method [30] (Bio-Rad, Hercules, CA). 50 µg of protein from cell extracts was fractionated on a 10% SDS-PAGE gel. After electrophoresis, proteins were transferred onto a nitrocellulose membrane in transfer buffer (192 mM glycine, 25 mM Tris-HCl, pH 8.8, and 20% methanol [v/v]) by electroblotting. After blocking with 5% skim milk for 1 h, the membrane was incubated with a 1∶1,000-diluted anti-His primary antibody (Santa Cruz Biotechnology, Santa Cruz, CA), washed three times with TTBS (50 mM Tris, 150 mM NaCl, and 0.5% Tween-20), and subsequently incubated with a peroxidase-conjugated anti-rabbit secondary antibody (GE Healthcare, Uppsala, Sweden) in milk containing TTBS for 1 h. After final washing, the membrane was then exposed and protein bands were detected using Enhanced Chemiluminescence (WESTSAVE GOLD; AbFrontier, Seoul, Korea).

### Cell Proliferation Assays (MTT Assay)

To assess the anti-proliferative activity of peptides and anti-Ras scFv fusion proteins, HCT116 cells (K-ras mutated cells) were seeded in 96-well plates at a density of 2×10^4^ cells/well in 100 µL of DMEM supplemented with 10% FBS and cultured for 24 h at 37°C. After 24 h of incubation, cells were treated with scFv or peptide-scFv fusion proteins (0, 0.5, 1 and 2 µM) and incubated for another 24 h. Cell viability was measured with the 3-(4,5-dimethylthiazol-2-yl)-2,5-diphenyl tetrazolium bromide (MTT) assay using the CellTiter 96® Non-radioactive Cell Proliferation assay kit (Promega, Madison, WI) according to the manufacturer’s instructions. The absorbance of the solution was measured at 570 nm using a Microplate Reader (Bio-Rad). Cell viability was expressed as the percentage of viable cells treated with scFv or peptide-scFv fusion proteins compared to the PBS-treated control (100%). All experiments were done in triplicate.

### Detection of Apoptosis

HCT116 cells (1×10^6^/well in a 6-well plate) were treated with peptide-fused scFvs (each, 2 µM) or a well-known apoptosis inducer staurosporine (0.5 µM) for 24 h at 37°C and cell extracts were prepared as described above. Cleaved poly (ADP ribose) polymerase (PARP), an indicator of apoptosis, was detected by Western blotting as described above. Anti-PARP and anti-α-tubulin antibodies (Cell Signaling Tech.) were used at a 1∶1,000 dilution as the primary antibodies, whereas horseradish peroxidase-conjugated anti-rabbit IgG (GE Healthcare, Uppsala, Sweden) was used at a 1∶10,000 dilution as the secondary antibody.

In addition, apoptotic cells were identified by staining with annexin V-FITC (150 ng/ml) and 7 amino-actinomycin D (7-AAD; BD Biosciences, San Diego, CA). 1×10^6^ HCT116 cells were treated with PBS, staurosporine (0.5 µM), or peptide-fused scFvs (each, 2 µM) as described above. At the designated time, cells were washed twice with PBS (pH 7.4), harvested and resuspended in 500 µl of binding buffer supplemented with Annexin-V fluos (1∶100 diluted; BioBud, Seoul, Korea), and incubated for 15 min at 25°C in the dark. To detect necrosis, 7AAD was added prior to measurement. Samples were immediately subjected to FACS analysis with a FACSCalibur flow cytometer (FL1 and FL3) and WinMDI software (Joe Trotter, Scripps Research Institute). 1×10^4^ cells per sample were analyzed by flow cytometry.

### Ras Activation Assay

To study the peptide-scFv fusion protein-dependent suppression of Ras activity, the level of Ras-GTP (active form) was measured using a Ras activation assay kit (Millipore, Billecia, MA) according to the manufacturer's instructions. This method is based on a selective binding of Ras-GTP using the Ras binding domain (RBD) of Raf-1 (a kinase downstream of Ras) that fails to bind Ras-GDP (inactive form). Briefly, 50 µl of RBD protein fused to glutathione transferase (GST) was coated on to a 96-well glutathione-coated plate at 4°C for 1 h and washed with TBST (50 mM Tris, 150 mM NaCl, and 0.05% Tween-20, pH 7.6). HCT116 cells were treated with scFv, Tat-scFv, or BR2-scFv for 1 or 2 h, after which cells were lysed using 1×Mg^2+^ Lysis/Wash Buffer (Millipore). The protein concentration was calculated by the Bradford assay. Cell lysates containing 50 µg of protein were incubated for 1 h in the RBD-GST-coated wells at 25°C. After washing three times with TBST, primary antibody solution was added and incubated at 4°C for 1 h. After washing with TBST, the secondary HRP-conjugated anti-mouse antibody was added and incubated at 25°C for 1 h. After a final washing with TBS (50 mM Tris, 150 mM NaCl, pH 7.6), 50 µl of the Chemiluminescent substrates was added to each well. Chemiluminescence signals from each well were monitored using the Berthold luminometer (MicroLumat LB96P; Berthold Technologies, Oak Ridge, TN). Results were expressed as relative Ras activity.

## Results

### BR2 Efficiently Internalizes into Various Cancer Cell Lines without Cytotoxicity to Normal Cells

**Figure 1:**
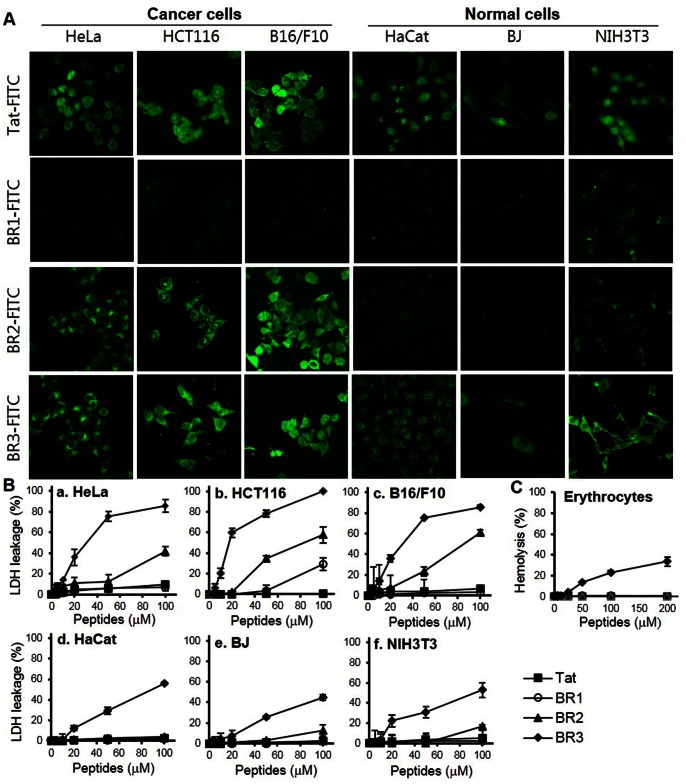
BR2 efficiently translocates into various cancer cells without cytotoxicity to normal cells. (A) Intracellular distribution of FITC-labeled peptides examined by confocal laser microscopy. Both cancer cells (HeLa, HCT116 and B16/F10) and normal cells (HaCat, BJ and NIH 3T3) were seeded in 6-well plates 1 day prior to the experiment to reach 70% confluence. Cells were incubated with FITC-labeled peptides (5 µM) for 30 min at 37°C and washed three times with phosphate buffered saline (PBS). Peptide distribution was then analyzed using a confocal scanning LSM 510 laser microscope equipped with a 40× objective. (B,C) *In vitro* cytotoxicity of peptides. (B) Membrane disturbance was measured by lactate dehydrogenase (LDH) leakage from the indicated cell lines 24 h after peptide treatment. LDH leakage from cells seeded in a 96-well plate at 10,000 cells/well was measured after exposure to 1, 2, 5, 10, 20, 50 or 100 µM peptides for 24 h. LDH release from PBS treated cells was regarded as 0% leakage and LDH released from 0.2% Triton X-100 treated cells as 100% leakage. (C) The hemolytic activity of each peptide against human erythrocytes was analyzed at graded concentrations (0–200 µM) and compared to a 0.2% Triton X-100 positive control, for which hemolysis was defined as 100%. Error bars in all figures represent the standard errors of the means (n = 3).

The cellular uptake and intracellular distribution of the designed peptides, BR1 and BR2, were studied using confocal microscopy. BR2 was readily internalized into cancer cells (HeLa, HCT116 and B16/F10) within 30 min and distributed throughout the cytoplasm and nucleus of cancer cells (Fig. 1A). However, it was very poorly internalized into normal cells (HaCat, BJ and NIH 3T3) under the same experimental conditions. In contrast, BR1 displayed negligible cellular uptake levels in both cancer and normal cells, indicating that it could not translocate across the cell membrane (Fig. 1A). Fluorescence from Tat-treated cells was also clearly detected in both the nucleus and cytoplasm of cancer and normal cells. More Tat accumulated in the nucleus than cytoplasm, whereas most BR2 was evenly distributed in the intracellular regions (Fig. 1A).

To assess the *in vitro* cytotoxicity of the designed peptides, the LDH leakage was measured in cancer cell lines (HeLa, HCT116 and B16/F10) and normal cell lines (HaCat, BJ and NIH 3T3). BR2 treatment (20–100 µM) was associated with LDH leakage from cancer cells; the leakage gradually increased with BR2 concentrations, reaching about 40–60% at 100 µM (Fig. 1B *a–c*). However, LDH leakage from the BR2-treated normal cells was not detected at BR2 concentrations below 70 µM, and a weak leakage (∼17%) was observed at 100 µM (Fig. 1B *d–f*). BR3 induced substantial LDH leakage from both cancer (approx. 80–90%) and normal cell lines (approx. 30%) even at 50 µM (Fig. 1B *a–f*).

To examine the cytotoxicity of peptides further, the hemolytic activity of BR1, BR2, BR3 and Tat against human erythrocytes was also examined. In accordance with the LDH leakage results, no hemolysis was induced even after the erythrocytes were treated with Tat, BR1 or BR2 at ≥200 µM, whereas BR3 triggered hemolysis (≥5%) at ≥25 µM (Fig. 1C). Subsequently about 34.3% of erythrocytes were lysed after being treated with 200 µM BR3. Taken together, these results indicate that BR2 efficiently penetrates cancer cells without cytotoxic effects in normal cells, whereas Tat showed similar penetration into both cell types.

### BR2 Specifically Penetrates Cancer Cells in a Concentration-dependent Manner

**Figure 2:**
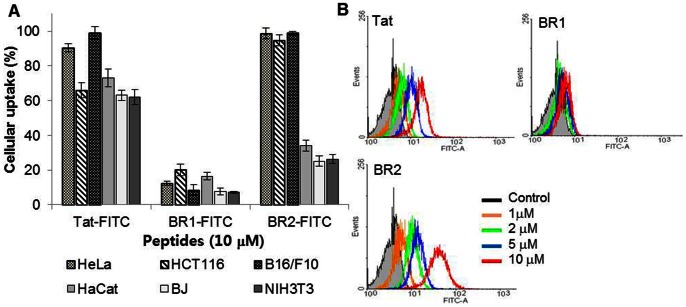
BR2 specifically penetrates cancer cell membranes in a concentration-dependent manner. (A) Analysis of the cell-penetrating efficiency of each peptide in different cell types by flow cytometry. FITC-labeled Tat, BR1 and BR2 peptides (10 µM) were added to different cell types: HeLa, HCT116 and B16/F10 cancer cells and HaCat, BJ and NIH 3T3 normal cells. After 30 min of incubation at 37°C, the FITC-positive cells were counted by flow cytometry. Values represent the percentage of fluorescence-positive cells in the total cell population. (B) Quantitative assessment of cell penetration of each peptide by flow cytometry. HeLa cells were incubated with FITC-labeled peptides at concentrations of 1, 2, 5, or 10 µM for 30 min at 37°C. Afterwards the cells were washed with cold PBS and harvested and cellular fluorescence was analyzed by flow cytometry. Control cells did not receive peptide treatment. Prior to analysis, extracellular fluorescence of surface bound peptides was removed by a trypsin treatment (1 mg/ml for 10 min).

To compare the quantity of the internalized peptides in different cell types, we performed flow cytometric analysis. BR2 was internalized more efficiently into cancer cells (HeLa, HCT116 and B16/F10) than into normal cells (HaCat, BJ and NIH 3T3); more than 95% of BR2-treated cancer cells internalized BR2, whereas only 23–34% of BR2-treated normal cells did. However, Tat penetrated both cancer and normal cells without much discrimination (Fig. 2A). Specific penetration of BR2 and Tat into cancer cells was also analyzed in the presence of both HeLa and BJ fibroblast cells by confocal laser microscopy. BR2 was preferentially uptaken by HeLa cells, whereas the cellular uptake of Tat was similar in both HeLa and BJ fibroblast cells (Fig. S1). These results clearly show that the buforin-derivative BR2 has cancer cell specificity, whereas Tat does not.

The fluorescence intensity of peptide-treated cells was enhanced with increasing concentrations of BR2 or Tat. At concentrations above 5 µM, BR2 entered more than 80% of HeLa cells within 30 min (Fig. 2B). BR2 penetrated cells more efficiently than did the Tat peptide at every tested concentration. Moreover, BR2 internalization for all cancer cell lines (HCT116 and B16/F10, data not shown) appeared to be homogeneous in the whole cell population as a single peak on the histogram. Among the peptides, BR1 displayed the lowest uptake, indicating its inability to penetrate the cell membrane even at 10 µM.

### Initial Electrostatic Interaction with Positively Charged BR2 and Negatively Charged Gangliosides on the Cancer Cell Membrane is Essential for the Energy-dependent Endocytosis of BR2

**Figure 3:**
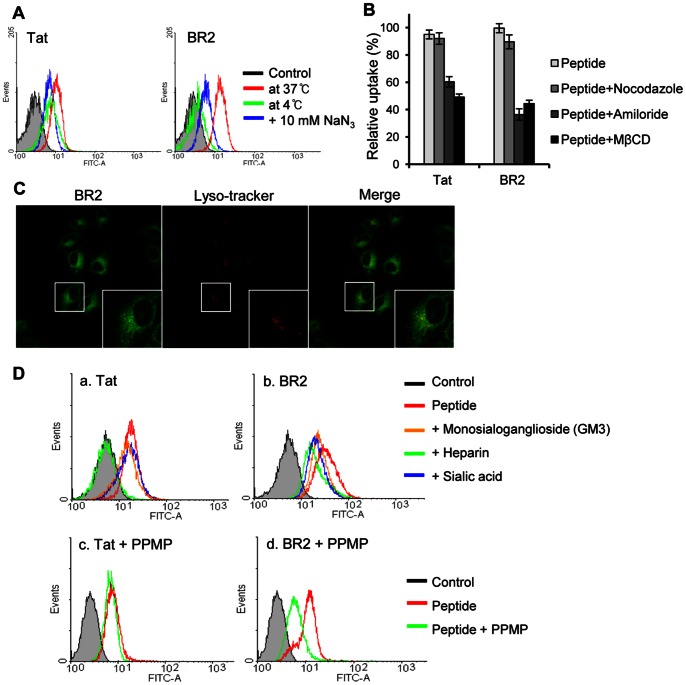
Contribution of energy-dependent pathways and negatively charged molecules on cancer cell membranes to peptide internalization. (A) Effects of low temperature and energy depletion on the internalization of FITC-labeled peptides into HeLa cells. HeLa cells were either preincubated at 4°C or pretreated with sodium azide (NaN_3_) to deplete ATP for 1 h, and then incubated with 5 µM Tat or BR2 for 30 min under the same conditions, as described in Materials and Methods. Peptide uptake was determined by flow cytometry. (B) Effects of endocytic inhibitors on the entry of BR2 and Tat. The influence of inhibitory drugs on peptide uptake was determined by preincubation of HeLa cells with the endocytosis inhibitors nocodazole, amiloride or methyl-ß-cyclodextrin for 1 h prior to the addition of FITC-labeled peptides. After peptide treatment for 30 min at 37°C, FITC-positive cells were counted by flow cytometry. Values represent the percentage of fluorescence-positive cells in the total cell population. Data represent the mean ± s.d. of three independent experiments. (C) Colocalization of BR2 with the lysosomal marker LysoTracker red DND-99 in living HeLa cells. After 30 min incubation of BR2 (5 µM) with LysoTracker, live HeLa cell images were obtained by confocal microscopy. (D) Effects of negatively charged molecules (gangliosides, heparins, and sialic acids) on peptide uptake. (a,b) HeLa cells were treated with BR2 and Tat in the presence of gangliosides, heparins, or sialic acids (each, 20 µg/ml) for 30 min. In (c,d), HeLa cells were pretreated with PPMP (5 µM) to deplete gangliosides. Cellular uptake of BR2 and Tat was determined by flow cytometry. All experiments were performed in triplicate.

The cellular uptake mechanism of BR2 was analyzed with HeLa, the most representative and widely used cancer cell line, to compare with those of other cell-penetrating peptides. We first examined the effect of temperature on BR2 penetration. Lowering the temperature dramatically reduced peptide uptake in HeLa cells (Fig. 3A); the cellular uptake of BR2 and Tat at 4°C was decreased by 88.5% and 31.6%, respectively, compared to that observed at 37°C. In addition, an energy-dependent uptake of peptides was also observed when the cellular ATP pool was depleted by preincubation of the cells with sodium azide (NaN_3_). ATP depletion also reduced the uptake of BR2 and Tat in HeLa cells, by 67.7% and 42.5%, respectively (Fig. 3A). These results support the involvement of endocytosis for BR2 and Tat uptake.

We next determined whether the cellular uptake of BR2 occurs through a specific endocytic pathway. Depletion of cholesterol from the plasma membrane with methyl-ß-cyclodextrin (MßCD) significantly inhibited the penetration of BR2 (44.6%) into HeLa cells, suggesting that BR2 penetrates the cell membrane via lipid raft-mediated endocytosis (Fig. 3B). Furthermore, pretreatment of cells with amiloride, a specific inhibitor of a sodium channel required for macropinocytosis, also prevented the cellular uptake of BR2 (36.5%). In contrast, pretreatment with nocodazole, an inhibitor of clathrin-mediated endocytosis, showed a negligible effect on peptide transduction. This result indicates that lipid raft-mediated macropinocytosis is a major mode of BR2 transduction.

To determine if BR2 is degraded via the lysosomal pathway, co-distribution with LysoTracker, an agent that accumulates in late endosomes/lysosomes, was examined. An overlay of respective images showed this lysosomal marker (red) primarily in a random punctuate distribution that was mostly separated from the BR2 signal (green) (Fig. 3C). BR2 was dispersed widely in the cytoplasmic region and the majority of BR2 was not colocalized with lysosomes. These results indicate that BR2 can escape from endosomes into the cytosol, bypassing further steps in the lysosomal pathway.

To identify the initial cell-surface binding targets for BR2 internalization into cancer cells, we examined whether exogenous gangliosides, heparins or sialic acids affected this process. Preincubation of gangliosides and sialic acids with BR2 partially inhibited peptide penetration into HeLa cells, whereas Tat penetration was not affected (Fig. 3D *a* and *b*). However, Tat uptake was severely reduced when heparin was added to the culture medium. Moreover, incubation of HeLa cells with the ganglioside synthesis inhibitor PPMP, significantly reduced BR2 but not Tat uptake (Fig. 3D *c* and *d*). These results indicate that gangliosides on cancer cell membranes are one of the main target molecules for BR2 binding.

### BR2-scFv Inhibits Cancer Cell Growth in a Dose-dependent Manner

The ability of BR2 to deliver proteins into cancer cells (HeLa cells) was demonstrated by fusion with an EGFP. BR2 delivered EGFP more efficiently into cancer cells than Tat (Fig. S2). These results suggest that BR2 can be used to deliver proteins efficiently into cancer cells by fusion with cargo proteins.

**Figure 4:**
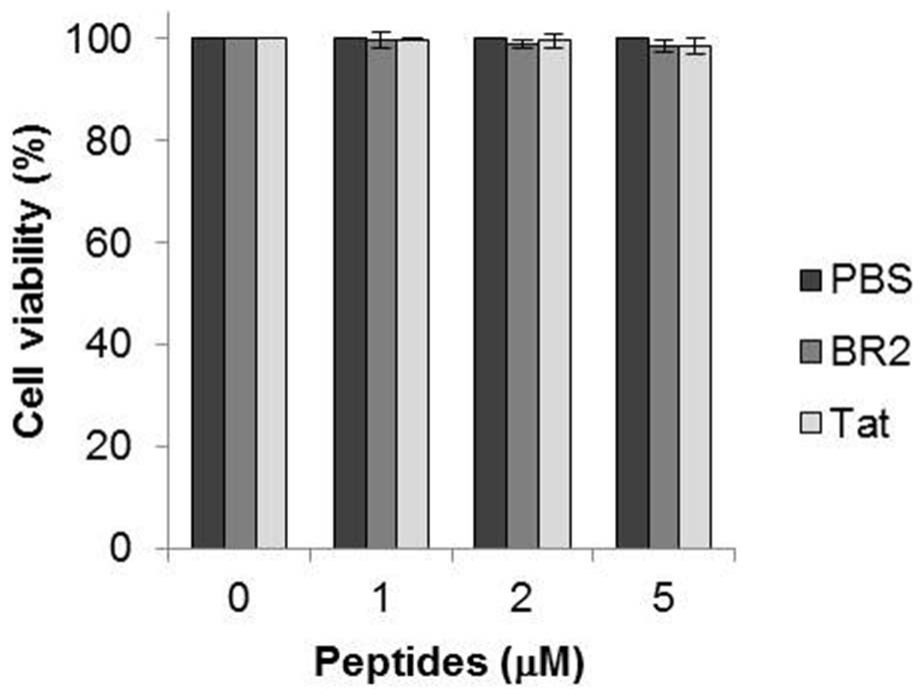
Intracellular uptake of peptides and anti-Ras scFv fusion proteins and their anti-proliferative activity. (A) Schematic representation of peptide-anti-Ras scFv cDNA constructs. DNA encoding peptides and the Y13-259-scFv cDNA were fused as described in Materials and Methods and cloned into the *Nco*I and *EcoR*I sites of pET21c. The white boxes represent V_H_ and V_L_ of the Y13-259 scFv sequence. The round box indicates the sequence encoding the peptides: Tat or BR2. The *Nco*I and *EcoR*I restriction sites and stop codon positions are also indicated. (B) Protein uptake was analyzed by Western blotting of fractionated lysates from HCT116 cells treated with PBS, anti-Ras scFv, BR2- or Tat-scFv fusion protein (each, 2 µM) at 37°C for 2 h. An anti-His antibody was used to detect intracellular Tat-scFv and BR2-scFv (28-kDa). (C) The anti-proliferative activity of peptides and anti-Ras scFv fusion proteins. HCT116 cells were exposed to the indicated concentrations of anti-Ras scFv, Tat- or BR2-scFv fusion protein at 37°C for 24 h. Cell proliferation was determined using the MTT assay. Data represent the mean ± s.d. of three independent experiments.

To further investigate the effects of BR2 on therapeutic protein delivery into cancer cells, Tat and BR2 were each fused with anti-Ras scFv (Fig. 4A), and the intracellular penetration of these fusions was assessed by Western blotting. This analysis showed that both Tat- and BR2-scFv fusion proteins were effectively delivered into HCT116 cells and accumulated in the intracellular region within 2 h, whereas the transduction of scFv itself was not observed (Fig. 4B). The amount of BR2-scFv fusion protein delivered into the cytoplasm was 32.6% more than that of the intracellularly delivered Tat-scFv fusion protein under the same experimental conditions.

Moreover, the intracellular localization of peptides and anti-Ras scFv fusion proteins in HCT116 cells was examined by immunocytochemistry analysis. The fluorescence was detected in cytoplasmic regions of Tat- and BR2-scFv treated cells (Fig. S3). Especially, BR2-scFv treated cells showed higher fluorescence intensity than Tat-scFv treated cells, indicating that BR2 delivered scFv more efficiently into cells than Tat did. Unlike Tat- or BR2-scFv fusion proteins, unconjugated scFv was not detected in the cells.

Next, we investigated the anticancer activity of peptide-scFv fusion proteins against Ras-mutated cancer cells by comparing it with the activity of unconjugated scFv *in vitro*. A significant reduction of cell viability was observed 24 h after the introduction of BR2-scFv or Tat-scFv fusion proteins versus unconjugated scFv (Fig. 4C), BR2 or Tat alone (Fig. S4). The viability of 2 µM BR2-scFv treated cancer cells was 39.3%, much lower than that of scFv or Tat-scFv treated cells, which had viabilities of 87.8% and 50.5%, respectively. Inhibition of cell proliferation positively correlated with protein concentration, suggesting that BR2-scFv more efficiently suppresses cancer cell proliferation than Tat-scFv.

### BR2-anti-Ras scFv Fusion Protein Promotes Apoptosis and Inactivates Ras in Ras-mutated Cancer Cells

**Figure 5:**
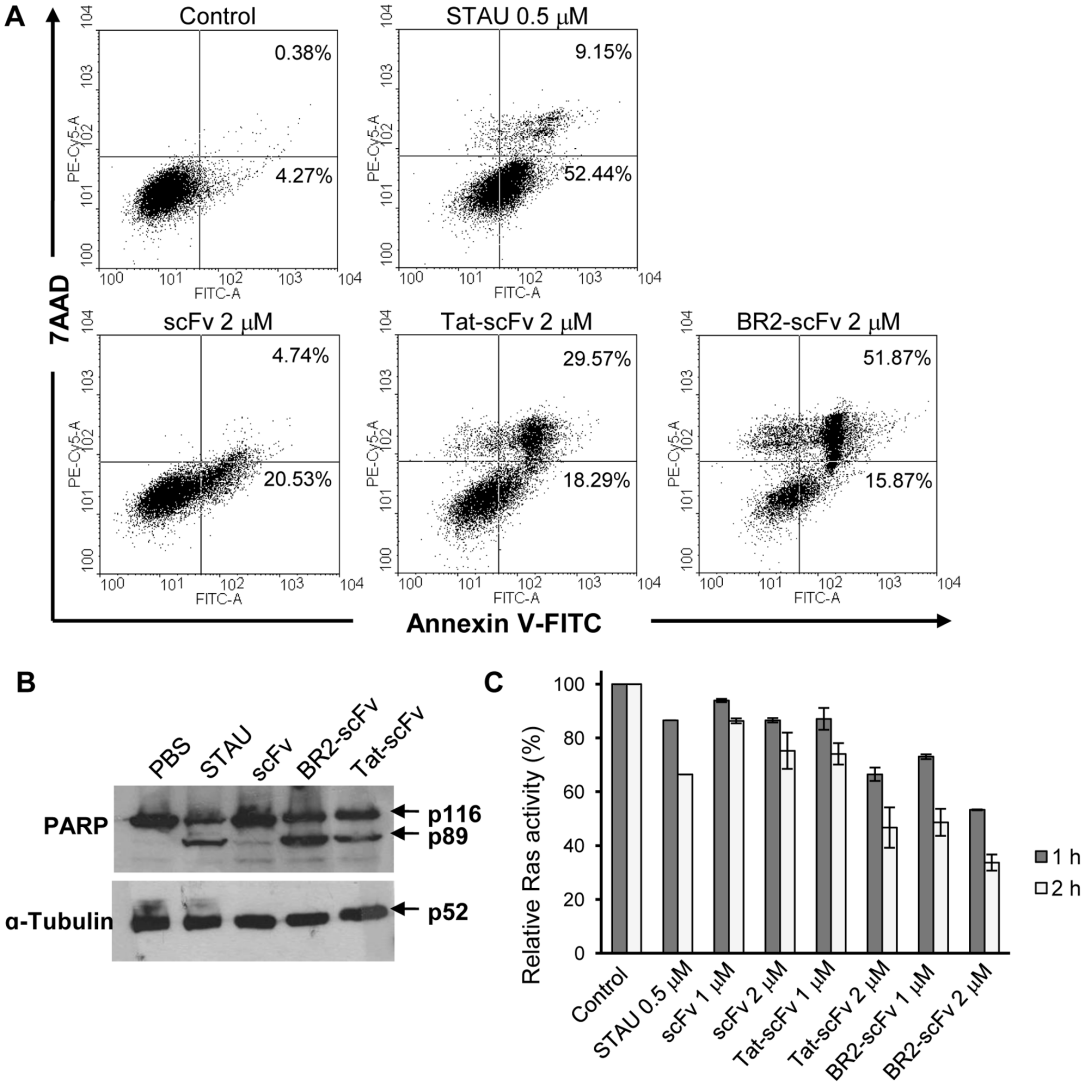
The BR2-anti-Ras scFv fusion protein promotes apoptosis by blocking Ras signaling in Ras-mutated HCT116 cells. (A) HCT116 cells were treated with anti-Ras scFv, Tat- or BR2-scFv fusion protein (each, 2 µM) or staurosporine (0.5 µM) for 24 h. Cells were stained with Annexin V-FITC and 7AAD to allow detection of apoptotic cell fractions by flow cytometry. The lower left quadrant contains the live cell (double negative) population; the lower right contains the apoptotic (annexin V+/7AAD-) population; the upper right contains the late apoptotic/necrotic (annexin V+/7AAD+) population; and the upper left contains the pre-necrotic (annexin V−/7AAD+) population. The numbers on the top of the quadrants indicate the percentage of apoptotic and late apoptotic/necrotic cells counted from dot plots taken from one representative experiment, performed in triplicate. (B) 24 h after PBS, anti-Ras scFv, Tat- or BR2-scFv fusion protein (each, 2 µM) or staurosporine (0.5 µM) treatment, HCT116 cell extracts were subjected to Western blot analysis with anti-PARP or anti-α-tubulin antibodies. The molecular sizes of the proteins are indicated with arrows at the right. α-tubulin is shown as a control. (C) Ras activation assay. Changes in Ras activity level in HCT116 cells treated with anti-Ras scFv and BR2- and Tat-scFv fusion proteins (each, 1 and 2 µM) were determined by an ELISA-based activity assay; results are expressed as relative Ras activity (%).

To examine how the BR2-scFv fusion protein inhibits cancer cell proliferation, we investigated whether apoptosis was induced. 67.7% of BR2-scFv treated cells and 47.9% of Tat-scFv treated cells were in the apoptotic stage, versus 5.6% and 25.3% of the PBS- and scFv-treated cells, respectively (Fig. 5A). The percentage of BR2-scFv treated cells that were apoptotic was higher than that of staurosporine-treated cells (61.6%). The apoptotic pathway induced by BR2-scFv was further investigated by Western blotting. Treatment of HCT116 cells with Tat- or BR2-scFv fusion proteins resulted in the cleavage of the 116-kDa PARP protein to the apoptosis-specific 89-kDa fragment after 24 h. A larger amount of the 89-kDa fragment was detected in the BR2-scFv treated cells as compared to the staurosporine- or Tat-scFv treated cells. However, the cleaved PARP fragment was not detectable in control cells or scFv-treated cells (Fig. 5B).

Next, we performed Ras activation assays that specifically detect activated GTP-bound Ras to obtain evidence for the suppression of Ras activity by peptide-scFv fusion proteins. These peptide-scFv fusion proteins and staurosporine reduced Ras activity similarly in HCT116 cells. As shown in Fig. 5C, a significant decrease of Ras activity in HCT116 cells was observed both 1 h and 2 h after peptide-scFv fusion protein treatment. The activity of Ras-GTP in cells was significantly suppressed by exogenous Tat- and BR2-scFv fusion proteins (1, 2 µM) in a dose- and time-dependent manner. Even though both Tat- and BR2-scFv fusion proteins induced a remarkable decrease in the levels of GTP-bound Ras, BR2-scFv was more effective than either staurosporine or Tat-scFv. The decreased relative Ras activity in cells treated with 2 µM Tat-scFv (about 34% and 53% after 1 h and 2 h, respectively) was roughly similar to that treated with 1 µM BR2-scFv (about 27% and 52% after 1 h and 2 h, respectively). Active Ras was decreased by about 67% when cells were treated with 2 µM BR2-scFv for 2 h (Fig. 5C).

## Discussion

Here, we report that BR2, which is derived from the anticancer peptide buforin IIb, has a potent ability to deliver therapeutic proteins specifically into target cancer cells. Buforin IIb is known for its efficient ability to penetrate cancer cells through an electrostatic interaction with gangliosides on the cancer cell surface [24]. Buforin IIb, however, is also cytotoxic to normal cells at high concentrations. Therefore, reducing this cytotoxicity is critical if buforin IIb is to be used for drug delivery. It has been reported by many researchers that hemolytic activity and cytotoxicity of α-helical peptides are closely correlated with their helicity; stronger helicity usually means the more complete non-polar face of an α-helical peptide, which is correlated with its higher apparent hydrophobicity when interacting with cell biomembrane, subsequently contributing to cell membrane lysis [31]–[33]. Therefore, we stepwisely reduced the helicity of buforin IIb to minimize hemolytic activity and cytotoxic effect of buforin IIb on normal cells while maintaining its cancer cell specificity. The stepwise elimination of the C-terminal RLLR repeats of buforin IIb results in cancer cell specific peptides with reduced cytotoxicity, BR1 and BR2.

Unlike BR1, BR2 transduced across the plasma membrane of various cancer cells and accumulated in the nucleus and cytoplasm within 30 min. We confirmed the autonomous translocation of BR2 into the cytoplasm with comparable efficiency in all cancer cell types investigated. BR2 penetrated cancer cell membranes more efficiently than the Tat peptide and in a concentration-dependent manner. Moreover, BR1 and BR2 did not show hemolytic activity even at 200 µM, whereas more than 30% hemolysis was induced by 200 µM BR3. Among the peptides tested, BR2 exhibited an efficient penetration into cancer cells without cytotoxicity to normal cells, whereas BR1 displayed a weak cell-penetrating ability and cytotoxicity. From these observations, we can conclude that the number of RLLR repeats at C-terminus affects the cell-penetrating ability and cytotoxicity of peptides. Two RLLR repeats are required for the efficient translocation of buforin-derivatives into cells; however, more than 3 repeats can cause gradual cell damage like that induced by buforin IIb.

Furthermore, we observed a considerable difference between the penetration of BR2 and Tat into normal cells. BR2 showed about 4-fold higher transduction efficiency into cancer cells versus normal cells whereas Tat showed similar penetration efficiency regardless of cell type. Possible reasons for why BR2 displays cancer cell specificity include distinctive features of the cancer cell membrane, such as different membrane composition, altered fluidity, more negative surface charges, higher transmembrane potential and an increased level of acidic components on the surface [34], [35]. It is also known that a cancer cell membrane typically contains a net negative charge due to a high expression of anionic molecules such as phosphatidyl serine (PS) and O-glycosylated mucins on the outer membrane leaflet [36]. To identify factors associated with cancer cell specificity of BR2 and analyze the cellular uptake mechanism of BR2, a mechanistic study was performed in HeLa cells, the representative cancer cell line. When we added negatively charged cellular membrane components, such as ganglioside or sialic acid, BR2 uptake was partially inhibited, indicating that exogenous gangliosides and sialic acids act as antagonists of the same molecules that bind BR2 and thereby hamper this binding. Depleting gangliosides with PPMP also decreased BR2 uptake. However, heparin, another negatively charged component, has almost no effect on BR2 binding to cancer cells. Prior to endocytosis, positively charged BR2 specifically interacts with negatively charged gangliosides on the cancer cell plasma membrane. In contrast, the high content of zwitterionic phosphatidylcholine in the outer membrane leaflet of normal cells confers an overall neutral charge to these cells, resulting in a reduced capacity for electrostatic interactions with BR2. This further decreases cell penetration of BR2 in normal cells.

CPPs are known to be internalized into cells by two different endocytic mechanisms [37], clathrin-dependent and clathrin-independent endocytosis [38]–[40]. Among several clathrin-independent pathways, lipid raft-mediated macropinocytosis, which gives rise to larger vesicles that do not necessarily fuse with early endosomes [41], is attributed to the internalization of BR2, as confirmed by the experiment using specific endocytosis inhibitors, such as amiloride, methyl-β-cyclodextrin, and nocodazole (Fig. 3B). Furthermore, the LysoTracker probes, which were used to track acidic organelle like lysosomes, revealed that most of internalized BR2s are present in the cytoplasm, but not in the lysosome, escaping lysosomal degradation. These results clearly show that BR2 can be an effective and long-lasting drug delivery vehicle.

Given the need for efficient therapeutic protein delivery systems, we investigated the possibility of using BR2 for delivering an anticancer therapeutic protein by fusing it with anti-p21 Ras Y13-259 scFv. Ras is a small GTP-binding protein that plays a critical role in the regulation of cell proliferation, transformation and differentiation. Over-expression or mutations in the Ras oncogene have been identified in a large number of human tumors (∼30%) and therefore constitute a primary target for cancer treatment [42]. It was reported that microinjection of the neutralizing anti-Ras monoclonal antibody Y13-259 into Ras-transformed rodent fibroblasts induces transient phenotypic reversion the cells *in vitro* and inhibits all biological responses that require Ras proteins [43], [44]. Moreover, intracellular expression of scFv, a fragment derived from antibody Y13-259, specifically promoted apoptosis in human cancer cells *in vitro* and led to tumor regression in a colon carcinoma tumor model *in vivo* [45]. Thus, in this study, we made recombinant proteins in which BR2 and Tat peptides were fused with anti-Ras scFv. These peptide-scFv constructs were efficiently internalized into K-ras mutated colon cancer HCT116 cells within 2 h. BR2 delivered anti-Ras scFv into the cells 32.6% more efficiently than Tat did, whereas peptide-unconjugated scFv was not detectable in the intracellular region.

BR2- and Tat-anti Ras scFv fusion proteins clearly exerted strong anti-proliferative activity in exponentially growing Ras-mutated cancer cells by inducing apoptosis, whereas BR2, Tat or unconjugated scFv did not show any detectable inhibitions at the same concentrations. It seems that BR2 and Tat contribute to the enhancement of intracellular delivery of conjugated scFvs without causing the cytotoxicity against HCT116. The better internalization efficiency of BR2-fused scFv in cancer cells may be the basis for the higher anticancer activity of this fusion protein as compared with Tat-scFv. Furthermore, inactivation of GTP-Ras proteins inside tumor cells might be mediated via neutralization by specific binding between Ras and BR2-scFv fusion proteins. Our results clearly suggest that BR2 promotes delivery and proper localization of fused scFv antibody fragments to their target antigen, the Ras protein. These results imply that after peptide-scFv fusion proteins penetrated the cell membranes, they specifically bound and neutralized the target mutant GTP-Ras proteins. All of these findings suggest that biologically active anti-Ras scFv can be efficiently introduced into target cancer cells by BR2.

In conclusion, we have found a novel CPP, BR2, which specifically penetrates cancer cells without causing cytotoxicity to normal cells. BR2 can be used to transport therapeutic proteins efficiently into target cells by covalent conjugation with the cargoes, which retain bioactivity. Although further studies are needed to assess the utility of using BR2 *in vivo* for delivering other kinds of therapeutics into target cancer cells as well as the feasibility of simple conjugations of BR2 to therapeutics for practical cancer therapy, the cancer-specific cell-penetrating peptide BR2 will provide valuable tools for the efficient cancer therapies.

## Supporting Information

Figure S1 **Cancer cell specific penetration of BR2.** Specific penetration into cancer cells of FITC-labeled Tat and BR2 were examined in the presence of both cancer and normal cells by confocal laser microscopy. HeLa and BJ fibroblast cells were seeded and co-cultured in the same well of a 6-well plate 1 day prior to the experiment to reach 70% confluence. Cells were incubated with FITC-labeled Tat or BR2 (5 µM) for 30 min at 37°C and washed three times with phosphate buffered saline (PBS). Nuclei were stained with Hoechst 33342 (blue). Peptide internalization was then analyzed using a confocal laser microscope. HeLa cells and BJ fibroblast cells were indicated with red arrows and white arrows, respectively.
(TIF)

Figure S2 **Efficient protein transduction mediated by BR2.** (A) Schematic representation of peptide-EGFP cDNA constructs. DNA encoding peptides (Tat, BR1 or BR2) and EGFP were fused as described in Supplementary Materials and Methods in Information S1 and cloned into the *Bgl*II and *Nde*I sites of pET16b. The *Bgl*II and *Nde*I restriction sites and factor Xa cleavage site are indicated. (B,C) Cellular uptake of peptide-EGFP fusion proteins was analyzed by confocal laser microscopy and flow cytometry. Purified EGFP or peptide-EGFP fusion proteins (10 µM) were incubated with HeLa cells at 37°C for 2 h.
(TIF)

Figure S3 **Intracellular localization of peptides and anti-Ras scFv fusion proteins using immunocytochemistry.** Intracellular localization of fusion proteins was analyzed in HCT116 cells by immunocytochemistry. HCT116 cells were incubated with scFv, Tat-scFv or BR2-scFv fusion protein (each, 2 µM) for 2 h at 37°C. Cells were washed with PBS, fixed and permeabilized. FITC-conjugated anti-His antibody was used to detect intracellular localization of scFv, Tat-scFv and BR2-scFv. Nuclei were stained with DAPI (blue). Intracellular localization of fusion proteins was then analyzed by confocal laser microscope.
(TIF)

Figure S4 **Cytotoxic effect of BR2 and Tat against HCT116 cells.** HCT116 cells were treated with PBS, BR2 or Tat (0, 1, 2 and 5 µM) and incubated for 24 h. Cell viability was measured by MTT assay. Data represent the mean ± s.d. of three independent experiments.
(TIF)

Information S1 (DOCX)
