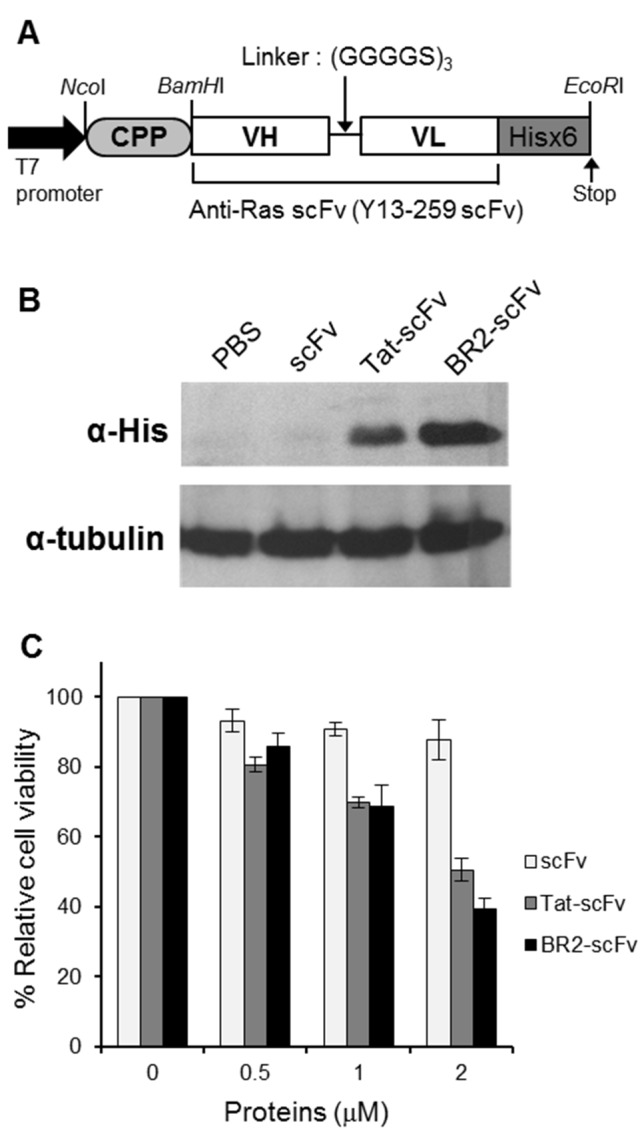# Correction: A Cancer Specific Cell-Penetrating Peptide, BR2, for the Efficient Delivery of an scFv into Cancer Cells

**DOI:** 10.1371/annotation/fb854e6a-cc9e-4446-b50a-5318cffb68c5

**Published:** 2013-11-15

**Authors:** Ki Jung Lim, Bong Hyun Sung, Ju Ri Shin, Young Woong Lee, Da Jung Kim, Kyung Seok Yang, Sun Chang Kim

Figure 4 is incorrect. Please see the correct Figure 4 here: 

**Figure:**