# Supplementary figures and images for: A Cancer Specific Cell-Penetrating Peptide, BR2, for the Efficient Delivery of an scFv into Cancer Cells
Source: PLoS One. 2013 Jun 11;8(6):e66084. doi: 10.1371/journal.pone.0066084 (PMC3679022; doi:10.1371/journal.pone.0066084)

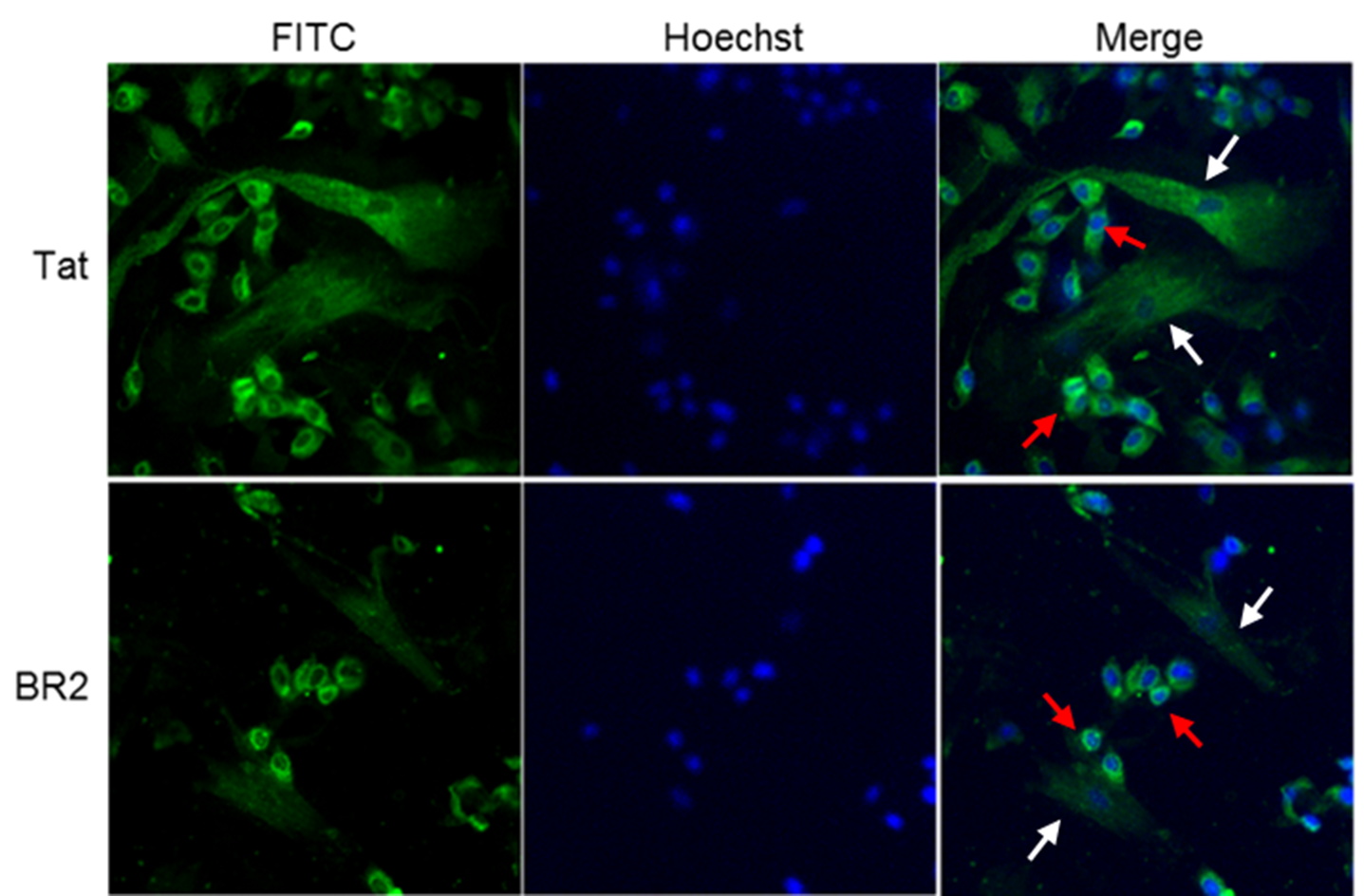

Supplement: Figure S1 — Cancer cell specific penetration of BR2. Specific penetration into cancer cells of FITC-labeled Tat and BR2 were examined in the presence of both cancer and normal cells by confocal laser microscopy. HeLa and BJ fibroblast cells were seeded and co-cultured in the same well of a 6-well plate 1 day prior to the experiment to reach 70% confluence. Cells were incubated with FITC-labeled Tat or BR2 (5 µM) for 30 min at 37°C and washed three times with phosphate buffered saline (PBS). Nuclei were stained with Hoechst 33342 (blue). Peptide internalization was then analyzed using a confocal laser microscope. HeLa cells and BJ fibroblast cells were indicated with red arrows and white arrows, respectively. (TIF) [file pone.0066084.s001.tif]

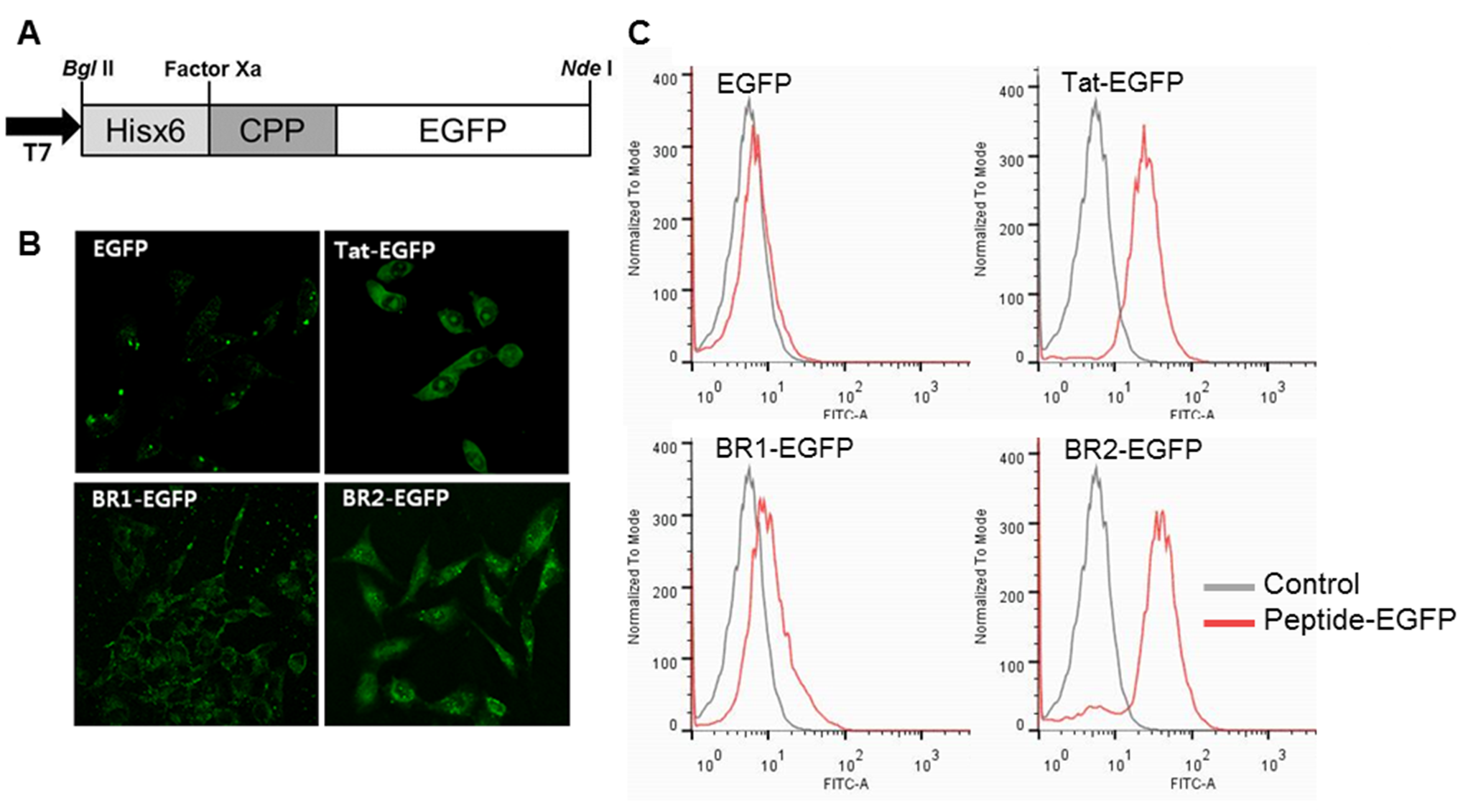

Supplement: Figure S2 — Efficient protein transduction mediated by BR2. (A) Schematic representation of peptide-EGFP cDNA constructs. DNA encoding peptides (Tat, BR1 or BR2) and EGFP were fused as described in Supplementary Materials and Methods in Information S1 and cloned into the BglII and NdeI sites of pET16b. The BglII and NdeI restriction sites and factor Xa cleavage site are indicated. (B,C) Cellular uptake of peptide-EGFP fusion proteins was analyzed by confocal laser microscopy and flow cytometry. Purified EGFP or peptide-EGFP fusion proteins (10 µM) were incubated with HeLa cells at 37°C for 2 h. (TIF) [file pone.0066084.s002.tif]

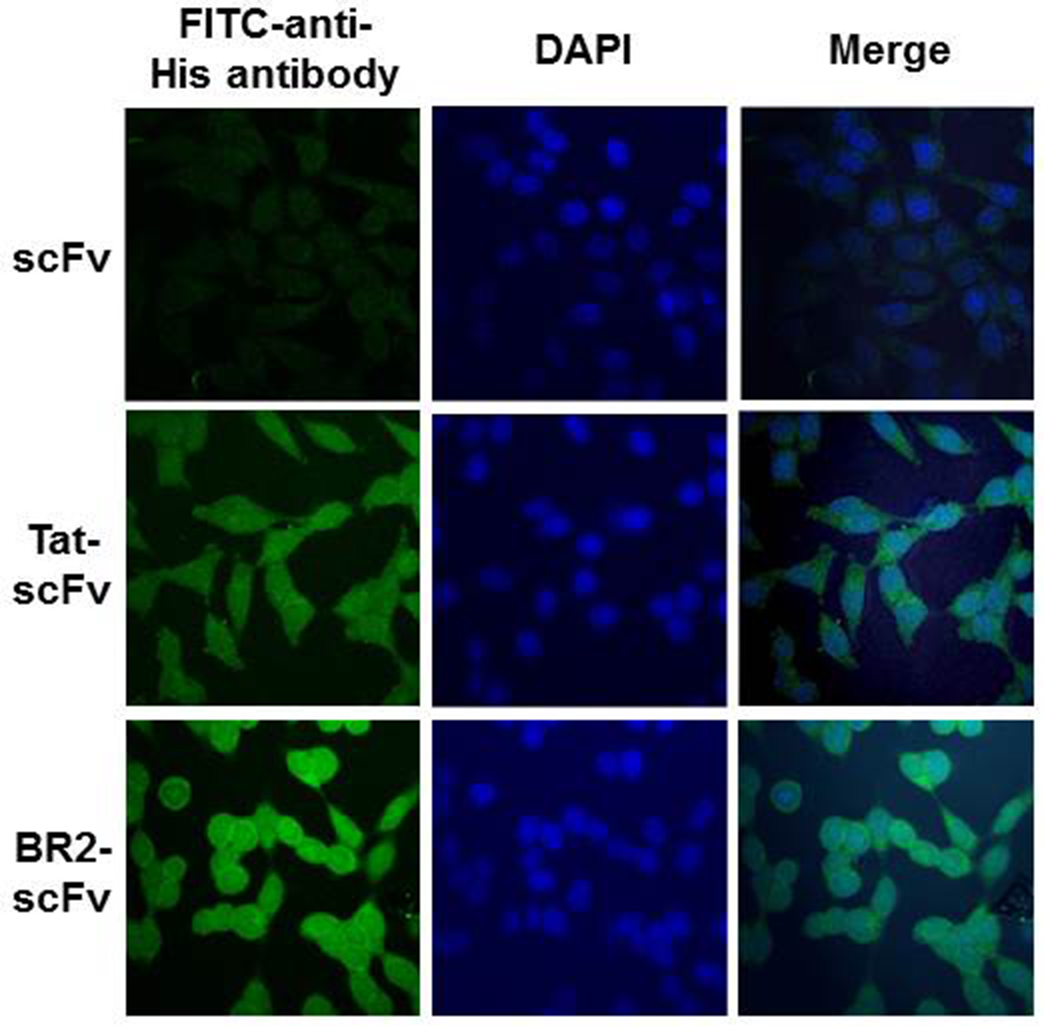

Supplement: Figure S3 — Intracellular localization of peptides and anti-Ras scFv fusion proteins using immunocytochemistry. Intracellular localization of fusion proteins was analyzed in HCT116 cells by immunocytochemistry. HCT116 cells were incubated with scFv, Tat-scFv or BR2-scFv fusion protein (each, 2 µM) for 2 h at 37°C. Cells were washed with PBS, fixed and permeabilized. FITC-conjugated anti-His antibody was used to detect intracellular localization of scFv, Tat-scFv and BR2-scFv. Nuclei were stained with DAPI (blue). Intracellular localization of fusion proteins was then analyzed by confocal laser microscope. (TIF) [file pone.0066084.s003.tif]

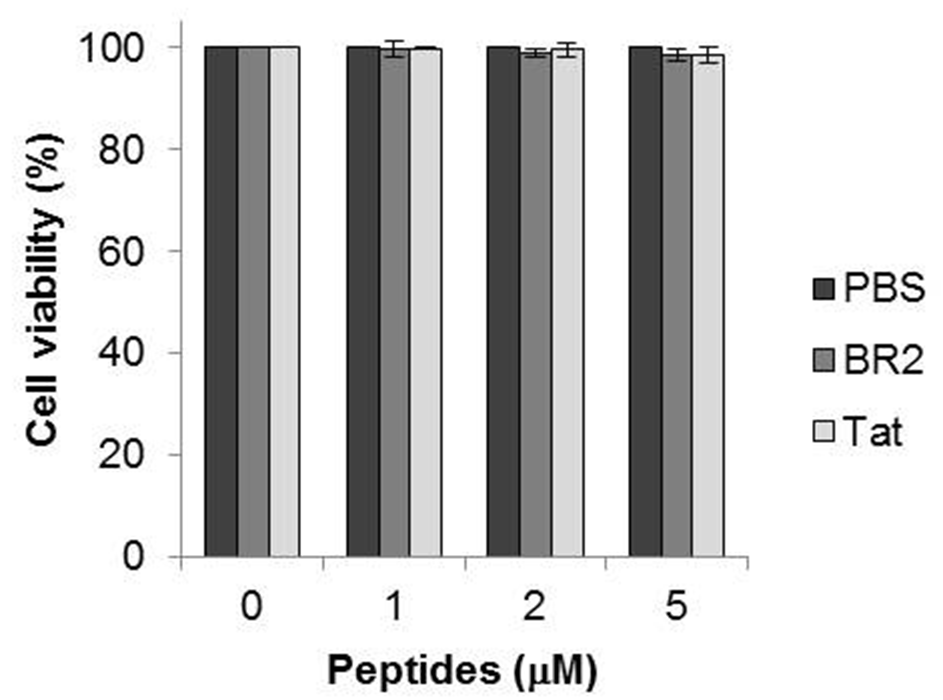

Supplement: Figure S4 — Cytotoxic effect of BR2 and Tat against HCT116 cells. HCT116 cells were treated with PBS, BR2 or Tat (0, 1, 2 and 5 µM) and incubated for 24 h. Cell viability was measured by MTT assay. Data represent the mean ± s.d. of three independent experiments. (TIF) [file pone.0066084.s004.tif]
