# Supplementary material for: A Cancer Specific Cell-Penetrating Peptide, BR2, for the Efficient Delivery of an scFv into Cancer Cells
Source: PLoS One. 2013 Jun 11;8(6):e66084. doi: 10.1371/journal.pone.0066084 (PMC3679022; doi:10.1371/journal.pone.0066084)
Supplement: Information S1 — (DOCX) [file pone.0066084.s005.docx]

**Supporting information**

**Supplementary Materials and Methods**

**Confocal laser scanning microscopy**

To investigate the cancer cell specific penetration of BR2, live confocal microscopy was performed in the presence of HeLa and BJ fibroblast cells. Each 1×10^5^ HeLa and BJ fibroblast cells were plated on the same glass coverslip placed in a 6-well plate, co-cultured for 24 h, and then incubated with FITC-labeled BR2 or Tat (5 μM, each) for 30 min. The nuclei were stained with 1 μg/ml Hoechst 33342 (Invitrogen, Carlsbad, CA). The cells were then rinsed three times with phosphate buffered saline (PBS, pH 7.4), and mounted on microscope slides with fluorescence mounting solution (Dako Corp, Carpinteria, CA). To avoid the effects of fixation artifacts, involving both methanol and paraformaldehyde, cells were not fixed (Lundberg *et al*., BBRC (2002); Richard *et al*., JBC (2003)). The intracellular distribution of FITC-labeled peptides was analyzed using a confocal scanning laser Zeiss LSM 510 microscope (Jena, Germany) equipped with a 20× objective. Fluorophores were excited at 488 nm for FITC and 346 nm for Hoechst 33342.

**Cloning and expression of the peptide-EGFP fusion proteins**

An enhanced green fluorescent protein (EGFP) encoding gene was obtained from a pEGFP-N1 (Clontech) by PCR and fused to C-terminus of each peptide (Tat, BR1, and BR2) by recombinant PCR. The recombinant genes were cloned into *Bgl*II and *Nde*I sites of pET16b (Novagen), producing pEGFP, pTat-EGFP, p-BR1-EGFP and pBR2-EGFP, respectively. EGFP, Tat-EGFP, BR1-EGFP and BR2-EGFP fusion proteins were expressed in *E. coli* BL21 (DE3) after induction with 0.1 mM IPTG for 8 h at 25°C. Cells were harvested by centrifugation at 3,000 × g for 15 min at 4°C and washed with 1 × PBS. The pellets were resuspended in His-tag binding buffer (20 mM Tris-HCl, 0.5 M NaCl, 5 mM imidazole, pH 7.9); cells were disrupted by sonication at 4°C (B. Braun instruments, Allentown, PA). The protease inhibitor phenylmethylsulfonyl fluoride (PMSF, 1 mM) was added prior to sonication. After centrifugation for 15 min at 14,000 × g at 4°C, supernatants were harvested and then passed through a Ni-affinity chromatography. The column was washed with wash buffer (20 mM Tris-HCl, 0.25 M NaCl, 60 mM imidazole, pH 7.9) and proteins were eluted with elution buffer (10 mM Tris-HCl, 0.25 M NaCl, 0.5 M imidazole, pH 7.9). His-tag of the purified proteins was removed by factor Xa protease digest (New England Biolabs, Beverly, MA) overnight at room temperature.

**Transduction studies of peptide–EGFP fusion proteins**

To investigate the protein delivery efficiency and the intracellular distribution of the internalized proteins, live confocal microscopy was performed on HeLa cells. Briefly, cells (2×10^5^) were plated on a glass coverslip, grown overnight, and then incubated with peptide-EGFP fusion proteins (each, 10 μM) for 2 h. The cells were then rinsed three times with phosphate buffered saline (PBS, pH 7.4), and mounted on microscope slides with mounting solution (Dako Corp, Carpinteria, CA). To avoid the effects of fixation artifacts, cells were not fixed (Lundberg *et al*., BBRC (2002); Richard *et al*., JBC (2003)). The distribution of peptide-EGFP fusion proteins was analyzed using a confocal scanning laser Zeiss LSM 510 microscope (Jena, Germany) equipped with a 20x objective. Fluorephores were excited with an argon laser (488 nm).

Quantification of the internalized peptide–EGFP recombinant proteins was measured in HeLa cells. Briefly, cells were seeded onto 12-well plates at a density of 2×10^5^ cells per well and incubated for 24 h. Peptide–EGFP fusion protein (10 μM, each) was incubated with the cells for 2 h at 37°C. Following the incubation, cells were washed three times with ice-cold PBS and trypsinized for 10 min to remove any remaining proteins bound to the cell surface. Cells were collected by centrifugation (1,000 × g for 5 min), resuspended with 500 μl ice-cold 2% FBS/PBS containing propidium iodide (PI), and then immediately analyzed (10,000 events/sample) by fluorescence activated cell sorting (FACS). Data acquisition and analysis were performed using the WINMDI software (Joe Trotter, Scripps Research Institute, La Jolla, CA). The statistical significance was evaluated by Student’s t-test at a 95% confidence interval.

**Immunocytochemistry**

To investigate the intracellular localization of peptide-scFv fusion proteins, immunocytochemistry was performed. HCT116 cells (2×10^5^) were plated on a glass coverslip placed in a 6-well plate, grown overnight, and then incubated with scFv, Tat-scFv or BR2-scFv (2 μM, each) for 2 h at 37°C. The cells were then washed three times with PBS, fixed with 4% (v/v) paraformaldehyde in PBS for 20 min at room temperature, and permeabilized with 0.1% Triton X-100 for 15 min at room temperature. After washing three times with PBS, cells were blocked with 3% BSA in PBS for 1 h at room temperature, and subsequently incubated with FITC-conjugated anti-His antibody (1:300 dilution in 1% BSA/PBS) (Santa Cruz Biotechnology, Santa Cruz, CA) for 2 h in dark. The cells were washed again five times with PBS, and then mounted on microscope slides with VectaShield mounting media containing DAPI (Vector Laboratories, Burlingame, CA). The intracellular localization of proteins was analyzed using a confocal scanning laser Zeiss LSM 510 microscope (Jena, Germany) equipped with a 40× objective.

**Cytotoxicity assay**

HCT116 cells were seeded in 96-well plates at a density of 2×10^4^ cells/well in 100 μl of DMEM supplemented with 10% FBS and cultured for 24 h at 37°C. After 24 h of incubation, cells were treated with Tat or BR2 (0, 1, 2 and 5 μM) and incubated for another 24 h. Cell viability was measured with the 3-(4,5-dimethylthiazol-2-yl)-2,5-diphenyl tetrazolium bromide (MTT) assay using the CellTiter 96^®^ Non-radioactive Cell Proliferation assay kit Promega, Madison, WI) according to the manufacturer’s instructions. The absorbance of the solution was measured at 570 nm using a Microplate Reader (Bio-Rad). Cell viability was expressed as the percentage of viable cells treated with Tat or BR2 compared to the PBS-treated control (100%). All experiments were done in triplicate.

**Supporting References**

1. Lee HS, Park CB, Kim JM, Jang SA, Park IY, et al. (2008) Mechanism of anticancer activity of buforin IIb, a histone H2A-derived peptide. Cancer Lett 271: 47-55.
2. Cho JH, Sung BH, Kim SC (2009) Buforins: histone H2A-derived antimicrobial peptides from toad stomach. Biochim Biophys Acta 1788: 1564-1569.
3. Shin SY, Lee SH, Yang ST, Park EJ, Lee DG, et al. (2001) Antibacterial, antitumor and hemolytic activities of α-helical antibiotic peptide, P18 and its analogs. J. Peptide Res. 58:504-514
4. Chen Y, Mant CT, Farmer SW, Hancock RE, Vasil ML, et al. (2005) Rational design of alpha-helical antimicrobial peptides with enhanced activities and specificity/therapeutic index. J Biol Chem. 280(13):12316-29
5. Huang YB, He LY, Jiang HY, Chen YX (2012) Role of helicity on the anticancer mechanism of action of cationic-helical peptides. Int J Mol Sci. 13(6): 6849-6862
6. Lundberg M, Johansson M (2002) Positively charged DNA-binding proteins cause apparent cell membrane translocation. Biochem Biophys Res Commun 291: 367-371.
7. Richard JP, Melikov K, Vives E, Ramos C, Verbeure B, et al. (2003) Cell-penetrating peptides. A reevaluation of the mechanism of cellular uptake. J Biol Chem. 278: 585-590.
8. Fredman P, Hedberg K, Brezicka T (2003) Gangliosides as therapeutic targets for cancer. BioDrugs, 17:155-67
9. De Coupade C, Fittipaldi A, Chagnas V, Michel M, Carlier S, et al. (2005) Novel human-derived cell penetrating peptides for specific subcellular delivery of therapeutic biomolecules. Biochem J. 390: 407-418
10. Duchardt F, Fotin-Mleczek M, Schwarz H, Fischer R, Brock R (2007) A comprehensive model for the cellular uptake of cationic cell-penetrating peptides. Traffic 8: 848-866
11. Kosuge M, Takeuchi K, Nakase I, Jones AT, Futaki S (2008) Cellular internalization and distribution of arginine-rich peptides as a function of extracellular peptide concentration, serum, and plasma membrane associated proteoglycans. Bioconjug Chemistry. 19: 656-664
12. Huang YB, Wang XF, Wang HY (2011) Studies on mechanism of action of anticancer peptides by modulation of hydrophobicity within a defined structural framework. Molecular Cancer therapeutics. 10: 416-426
